# Improving patient care for attention deficit hyperactivity disorder in children by organizational redesign (Tornado program) and enhanced collaboration between psychiatry and general practice: a controlled before and after study

**DOI:** 10.1186/s13012-014-0155-3

**Published:** 2014-10-30

**Authors:** Mijnke Janssen, Michel Wensing, Rutger Jan van der Gaag, Ineke Cornelissen, Patricia van Deurzen, Jan Buitelaar

**Affiliations:** Karakter Child and Adolescent Psychiatry University Centre, Reinier Postlaan 12, Nijmegen, 6525 GC The Netherlands; Scientific Institute for Quality of Healthcare, Radboud University Medical Centre, Nijmegen, The Netherlands; Department of Cognitive Neuroscience, Donders Institute for Brain, Cognition and Behaviour, Radboud University Medical Centre, Nijmegen, The Netherlands; Department of Psychiatry, Radboud University Medical Centre, Nijmegen, The Netherlands

**Keywords:** ADHD in children and adolescents, ADHD in child and adolescent psychiatry, ADHD in primary care, Implementation science, Organizational redesign

## Abstract

**Background:**

Implementation of clinical guidelines for diagnosis and treatment of attention deficit hyperactivity disorder (ADHD) in children and adolescents is a challenge in practice due to insufficient availability of mental health specialists and lack of effective cooperation with primary care physicians. The Tornado program aims to reduce time between referral and start of treatment in eligible patients. This study aims to assess the effectiveness and efficiency of this program.

**Methods/design:**

This is a non-randomized controlled before-after study involving 90 outpatients (6-18 years old) suspected of uncomplicated ADHD, which were recruited by ten mental health teams. The Tornado program, provided by three teams, combines accelerated-track diagnosis and treatment planning. This is followed by psychoeducation at a mental health center and pharmacological treatment by primary care physicians, who received an online e-learning module for this purpose. The control group consists of patients of seven other teams, who receive care as usual. Primary outcome is the patients' time between referral to the mental health or pediatric center and start of treatment. Secondary outcomes include severity of ADHD symptoms; functional status; health-related quality of life; treatment adherence; indicators of diagnostic procedures and treatments; patient, parent, and professional experiences and satisfaction with care; and an economic evaluation. The study is powered to detect a difference of 36 days.

**Discussion:**

This study will provide insight into the effectiveness and efficiency of the Tornado program, an accelerated-track program in mental healthcare.

**Trial registration:**

Netherlands Trial Register http://www.trialregister.nl/trialreg/admin/rctview.asp?TC=2505. Trial status: active data collection.

**Electronic supplementary material:**

The online version of this article (doi:10.1186/s13012-014-0155-3) contains supplementary material, which is available to authorized users.

## Background

Attention deficit hyperactivity disorder (ADHD) is a common neurodevelopmental disorder with an estimated worldwide prevalence of about 5% in children and adolescents. ADHD is characterized by an enduring pattern of inattention, hyperactivity, and impulsiveness and has a high persistence over adolescence into adulthood [1]. In the majority of patients, ADHD is complicated by the presence of comorbid disorders. A challenge, however, is to get patients quickly into adequate treatment after detection and referral to mental health care.

The most often offered evidence-based treatment for children and adolescents with ADHD is prescription of immediate-release or extended-release psychostimulants or non-stimulant medication (atomoxetine). Medication treatment is mostly preceded by psychoeducation and maybe combined with behavioral parent training, which is recommended by evidence-based clinical guidelines [2]. The various ADHD guidelines advise differently on the preferred order of available evidence-based treatment and which professional is to provide this care. The National Institute for Health and Care Excellence (NICE) does not recommend starting medication for children and adolescents with mild ADHD and it adopts a very clear vision that primary care providers should refer patients for diagnosis or start treatment [3]. The American Association of Child and Adolescent Psychiatry (AACAP) recommends starting medication, preceded by psychoeducation and when needed combined with behavioral treatment. In the guideline, the AACAP speaks of `clinicians' as the central caregivers without further specification [4]. The American Association of Pediatrics recommends starting medication and/or evidence-based parent- and/or teacher-administered behavior therapy as treatment for ADHD, preferably both. The primary care physician plays the central role in diagnosing and treating ADHD in children and adolescents in this guideline. The AAP considers relegating mental health conditions exclusively to mental health clinicians a non-viable solution for many clinicians, because in many areas, access to mental health clinicians to whom primary physicians can refer patients is limited [5]. The Dutch national multidisciplinary guideline for the assessment and treatment of ADHD in children and adolescents recommends starting medication, preceded by psychoeducation and when needed combined with behavioral treatment. They advise ADHD to be diagnosed and treated by secondary care mental health specialists (child psychiatrists and psychologists) and pediatricians [6].

There are several problems in daily practice for the implementation of these recommendations, resulting in delayed start of recommended treatment for many patients. Large numbers of children referred to specialist care result in long waiting lists in mental healthcare [7]. Many primary care physicians perceive pressure to make an initial diagnosis and start treatment. For instance, parents sometimes are being urged by the school of their child to seek clinical referral and medication treatment to safeguard placement of their child in the regular school system. Overall, primary care physicians are involved in about half of all ADHD cases where medication is given; they start medication in between 6% and 20% of all such cases [7]. Primary care physicians write 61% of the repeat prescriptions methylphenidate of children in the Netherlands. In 20% of these cases, no systematic follow-up is done [8]. Over diagnosing of ADHD as well as inadequate medical treatment and a shortage of systematic aftercare are well-known problems in primary care [9],[10]. Most primary care physicians consider themselves not sufficiently competent to take over the medication treatment of children and adolescents who first have been successfully regulated by the medical specialist. They miss synchronization of the cooperation between them and professionals of the second and third line. There is a lack of not only instructions for referral when ADHD is suspected but also of instructions for monitoring ADHD medication after interventions in the second of third line (aftercare). Collaboration arrangements and distribution of tasks between professionals differ vastly per region, probably caused by the availability of the various disciplines [11]. Furthermore, not all pediatricians diagnose and treat children and adolescents with ADHD.

In other countries as well, new ways for effective organization of the diagnostics and care for child and adolescent mental health are being proposed and studied. For instance, in the UK, a systematic review concluded some preliminary evidence that treatments by specialist staff working in primary care were effective, although the quality of included studies was variable and no data were available on the cost-effectiveness of interventions. Equally, some educational interventions showed potential for increasing the skills and confidence of primary care staff, but controlled evaluations were rare and few studies reported actual changes in professional behavior or patient health outcomes. A significant program of research was recommended if the potential for child and adolescent mental health services in primary care is to be realized in an effective and efficient way [10]. The Tornado program study builds on this previous study.

We felt that neither quick increase of the psychiatric work force nor nationwide education of primary care physicians would be feasible and efficient approaches to better implementation of the ADHD guideline. Although the guidelines advise no mediation treatment to be conducted by primary care physicians, good and practical methods to involve primary care physicians in the treatment of children and adolescents with uncomplicated ADHD is important for several reasons. Primary care physicians already have a lot of experience with the treatment and structures guidance of patients with a variety of psychiatric disorders and other chronic diseases. They are also familiar with the care for entire families. When family doctors could play a more important role in the treatment of uncomplicated ADHD of children and adolescents, the mental health specialist would be able to focus more on patients with complicated ADHD, for example, those with other psychiatric problems (44%) [10].

The Tornado program makes an effort to combine evidence-based guidelines with a more practical approach to organize the care for children and adolescents with uncomplicated ADHD. In this program, the diagnoses are made by secondary and tertiary line specialists, as proposed in the 2005 Guideline; the risk for under- and over diagnosing is therefore probably diminished. The medication (methylphenidate) will be prescribed and monitored according to guideline recommendations, due to the targeted education for general practitioners in how to prescribe and monitor ADHD medication. This education will also probably have positive effects on primary care physicians' feeling of competence regarding prescribing and monitoring medication for ADHD. The division of patients between first, secondary, and tertiary diagnosing and treatment facilities will be more balanced, resulting in a diminishing of the waiting lists of the youth mental health institutions.

### Aim of this study

The aim of the Tornado program is to shorten the patients' time between referral and start of recommended treatment by a one-day-to-diagnose service in mental healthcare and tailored professional education to facilitate the involvement of primary care physicians. The presented study aims to determine the effectiveness and efficiency of the Tornado program for uncomplicated ADHD in children and adolescents compared to usual care.

The key objectives are:

1. Examine the effect of the Tornado ADHD implementation program on time between referral from primary care and start of the treatment in mental healthcare, compared to usual care.
2. Examine the effectiveness regarding ADHD symptoms, functional status, health-related quality of life, treatment adherence, client experiences, guideline adherence of the professional, costs, and utilities, compared to care as usual.

Additional research questions are:

1. How do the professionals perceive and evaluate the program, particularly with respect to referral process (forth and back) between the general practitioners and specialists, compared to usual care?
2. Do the primary care physicians involved in this program believe they are competent to prescribe and monitor ADHD medication? How do they evaluate the online course about prescribing and monitoring medication for ADHD?

## Study design

This is a non-randomized pragmatic evaluation with a comparative before-after design. The clusters comprise ten treatment teams at nine different locations. Given the requirements of organizing the Tornado program, it was not possible to allocate the program randomly to treatment teams.

This study received approval from the Medical Ethics Committee of the Radboud University Medical Centre, Nijmegen, the Netherlands.

### Participants

The caregivers' population consists of primary care physicians, psychiatrists, psychologists, and pediatricians involved in diagnostics and/or treatment of children and adolescents with ADHD in the provinces Gelderland and Overijssel and the east of Noord-Brabant in the Netherlands.

The patient population consists of children and adolescents (6-18 year-olds) referred with suspicion of uncomplicated ADHD to a child mental health center or pediatrician. ADHD patients with psychiatric comorbidity and/or family problems that required clinical interventions for comorbidity and/or family problems were considered to be complicated ADHD and were excluded from the study. Directly after referral to mental healthcare, they are invited to participate. Their parents/caregivers are also involved in the study, as well as the involved professionals (GPs, psychiatrists, pediatricians, secretaries) of the Tornado ADHD program and CAU.

Characteristic of the (sub-)group of patients, targeted in this proposal, is the uncomplicated (no severe comorbidity and/or severe family problems) attention deficit hyperactivity disorder (ADHD) in children and adolescents (6-18 year-olds).

Patients are recruited in mental health centers. Eligibility criteria for the centers are treatment facility for children and adolescents with uncomplicated ADHD, and the included centers represent a variability of secondary healthcare centers involved in usual ADHD care in the Netherlands. The centers vary in yearly number of attending patients. Eligibility criteria for care providers on the locations are a professional qualification (child and adolescent psychiatrist, pediatrician, mental healthcare psychologist) and experience in the diagnostics and/or treatment of ADHD. Eligibility criteria for the general practitioner are the location of the practice.

### Interventions to be implemented

As recommended by the multidisciplinary ADHD guideline [6], the following procedures are implemented in the Tornado program:

1. Triage of referred patients to confirm the suspicion of uncomplicated ADHD.
2. Compact procedures for diagnostic process (shorter than current usual care).
3. Start of appropriate medication treatment.
4. Systematic monitoring and follow-up of the treatment results.
5. Focused parent training in four sessions in the treatment center.
6. Tailored professional education for primary care physicians who have referred a patient.

Each primary care physician who refers a patient to the Tornado ADHD program will be invited to fulfill the accredited e-learning module on ADHD for GPs before the treatment of his/her first enrolled patient starts.

### Implementation strategy: Tornado program

In the ADHD Tornado program, the registration coordinator of the psychiatric outpatient clinic selects patients with presumably uncomplicated ADHD on referral. The primary care physician of these patients is invited to participate in a tailored one-hour online-accredited course with information about ADHD and the prescription and monitoring of methylphenidate by the primary care physician. Children and their parents are informed about the proposed short diagnostic process of this program and the fact that the medication (when advised after intake) will be prescribed and monitored by their primary care physician.

When the primary care physicians, parents, and patients older than 10 years old, agree to participate in this program, a one-day diagnostic assessment is executed in a psychiatric outpatient clinic for children and adolescents. At the same time, the outpatient clinic invites the primary care physicians to participate in a one-hour online course about ADHD. The main themes of this course are information about the characteristics of ADHD and how to start methylphenidate and monitor and deal with the (side) effects of this medication. When patients return to their primary care physician with the diagnosis uncomplicated ADHD and a treatment advice, the primary care physician starts and monitors the methylphenidate. Parents receive psychoeducation in the outpatient clinic.

#### Control condition

Usual care exists of a standard diagnostic assessment following the Dutch guidelines, medication treatment (methylphenidate) when indicated after diagnosing. This medication is started and continued by the medical specialist. Psychoeducation for parents is provided within secondary care.

The Tornado program is implemented in three treatment teams: two teams in a specialized center for child and adolescent psychiatry and one team in a mental healthcare center. The control condition with usual care consists of six outpatient child and adolescent psychiatric clinics and one pediatric clinic.

### Measures

The key aim of the Tornado ADHD program is a reduction of time between referral and start treatment for the patients. The primary outcome of the study is time between referral to the mental health center and start of treatment. We will examine the time between referral and start treatment (T1 and T4, see Table 1).

**Table 1:** **Participant flow, time points, questionnaires, and respondents**

	Study group Tornado***n***= 90			Control group care as usual***n***= 90		
Timepoints	Child (if ≥ 10 years)	Parents/caregivers	Professional (psychiatrist (T2) and general practitioner (T4 and T7))	Child (if ≥ 10 years)	Parents/caregivers	Professional (psychiatrist or pediatrician)
*T1 = referral*	HoNOSCA	ADHD-RS		HoNOSCA	ADHD-RS	
	Kidscreen	HoNOSCA		Kidscreen	HoNOSCA	
	EQ-5D	Kidscreen		EQ-5D	Kidscreen	
		EQ-5D			EQ-5D	
		Extra questions^a^			Extra questions^a^	
*T2 = intake and start diagnostic process*	HoNOSCA	ADHD-RS	ADHD-RS	HoNOSCA	ADHD-RS	ADHD-RS
	Kidscreen	HoNOSCA	CGI	Kidscreen	HoNOSCA	HoNOSCA
		Kidscreen	HoNOSCA		Kidscreen	CGI
		EQ-5D			EQ-5D	
*T3 = consultation and end of diagnostic process*	GGZ-thermometer	GGZ-thermometer		GGZ-thermometer	GGZ-thermometer	
*T4 = start pharmacotherapy*	HoNOSCA	ADHD-RS	ADHD-RS	HoNOSCA	ADHD-RS	ADHD-RS
	Kidscreen	HoNOSCA	CGI	Kidscreen	HoNOSCA	HoNOSCA
		Kidscreen			Kidscreen	CGI
		Tic-P			Tic-P	
		EQ-5D			EQ-5D	
*T5 = 6 weeks after start pharmacotherapy*	HoNOSCA	ADHD-RS		HoNOSCA	ADHD-RS	
	Kidscreen	HoNOSCA		Kidscreen	HoNOSCA	
	Morisky	Kidscreen		Morisky	Kidscreen	
		Morisky			Morisky	
		EQ-5D			EQ-5D	
*T6 = 9 months after T1*	Morisky	Morisky		Morisky	Morisky	
	HoNOSCA	HoNOSCA		HoNOSCA	HoNOSCA	
	Kidscreen	Kidscreen		Kidscreen	Kidscreen	
	EQ-5D	EQ-5D		EQ-5D	EQ-5D	
*T7 = 1 year after T0*	HoNOSCA	ADHD-RS	ADHD-RS	HoNOSCA	ADHD-RS	ADHD-RS
	Kidscreen	HoNOSCA	HoNOSCA	Kidscreen	HoNOSCA	HoNOSCA
	EQ-5D	Kidscreen	CGI	EQ-5D	Kidscreen	CGI
	Morisky	Tic-P		Morisky	Tic-P	
	GGZ-thermometer	EQ-5D		GGZ-thermometer	EQ-5D	
		Morisky			Morisky	
		GGZ-Thermometer			GGZ-thermometer	

^a^Sex, family constitution, education level parents.

Clinical outcomes have been included as secondary outcomes, in the expectation that effects on mental health status and symptoms will not be different between the two study groups. The secondary outcomes are:

1. ADHD symptom severity. The clinical indicator measuring the ADHD symptoms is the ADHD Rating Scale (ADHD-RS). The ADHD-RS is a 12-item instrument that uses observer ratings (parent and caregiver) and self-report ratings to help assess attention deficit/hyperactivity disorder (ADHD) in children and adolescents. Each item is rated on a three-point scale. This rating scale is used in clinical and in research setting to establish a baseline measurement and monitor treatment effectiveness and changes over time [12],[13].
2. Functional status. The Health of the Nation Outcome Scale (HoNOSCA) provides a global measure of an individual's current mental health status and thus provides a means of evaluating the success of attempts to improve the health and social functioning of mentally ill children and adolescents [14],[15]. Although the clinician-rated HoNOSCA is the principal measurement tool, self-rated and parental-rated versions of HoNOSCA have also been developed and will be used in the present study.
3. Health-related quality of life. The Kidscreen-10 is a parent- and self-report questionnaire with ten items to assess global health-related quality of life for monitoring use [16].
4. Treatment adherence. The Morisky Adherence Scale provides a brief screening of adherence with treatment [17]. This scale has four items with dichotomous (yes/no) response options. The sum of 'yes' responses provides a total score of non-adherence. The scale has been used extensively among patients with varying medical conditions, including psychiatric disorders.
5. Patients' and parents' experiences and satisfaction. Parents and patients are asked about their experiences and satisfaction, using the Trimbos thermometer [18]
6. Professionals' experiences. We will conduct semi-structured telephone interviews with primary care physicians in the study group to assess their experiences with the Tornado program. The focus of these interviews will be on:
  Their knowledge and experience in diagnostics and treatment of children with ADHD.
  Their opinion and experience with respect to the referral process (forth and back), compared to usual care.
  Do the involved primary care physicians feel themselves competent enough to prescribe and monitor ADHD medication?
  Do the primary care physicians involved in the ADHD program think that the online course about prescribing and monitoring medication for ADHD is effective? Is there a difference in their feeling of competence before and after the course?
7. We will also conduct semi-structured interviews with the professionals involved in the Tornado ADHD implementation program with particularly respect to the referral process between general practitioners and specialists, compared to care as usual.
  Adherence of care providers to the protocol will be assessed by checking the response rates of care providers on the implemented symptom monitoring checklists. Further, a random sample of patient files will be checked to assess the medication protocol adherence of care providers.
8. Utilities. The EQ-5D measures utilities consists of a descriptive system and a visual analog scale (VAS) [19]. The descriptive system comprises five dimensions: mobility, self-care, usual activities, pain/discomfort, and anxiety/depression. Each dimension has three levels of perceived problems: no problems, some problems, and severe problems. Subsequently, the respondent is asked to self-rate the state of health on a vertical analogue scale. The VAS ranges from 0 to 100, where 100 is rated as `Best imaginable health state' and 0 as 'Worst imaginable health state'.
9. Costs (healthcare consumption and productivity loss). The TiC-P is a questionnaire to collect data on healthcare consumption and productivity losses [20]-[22].

Table 1 illustrates the participant flow, the time points, the questionnaires, and the respondents.

Sample size calculation:

The sample size calculation is based on real data from the outpatient clinic in Nijmegen, in which the Tornado program was piloted. The primary outcome of this study is time between referral and start of treatment. This time period was, on average, 67 days in usual care and 31 days in the Tornado program. So, the expected gain on this outcome is 36 days, with a standard deviation (SD) of 60 days, an intra-cluster correlation coefficient (ICC) of 0.03, an alpha of 0.05, and power set at 0.80. This implies that a total of six treatment teams is needed (three Tornado teams and three care as usual teams), which each provide data on 27 patients (*n* = 81 per study group). We anticipate on drop-out of participants and plan to include 90 patients in the intervention group and 90 patients in the control group.

#### Blinding

The care providers, the participants, and the parents are all outcome assessors and are not blinded to group assignment. Data analysis will be blinded to group assignment.

#### Statistical methods

The study will be performed and reported according to the published CONSORT recommendations for cluster trials. This implies, among others, an intention to treat approach as primary analysis. In case of missing data, multiple imputation of missing values will be performed. Clustering in the data within treatment teams will be taken into account by the use of random coefficient regression models. Subgroup analyses will be reported as explorative analyses.

The primary analysis will be a regression analysis of the primary outcome on study group (intervention versus control), controlling for prognostic patient factors' symptom severity at baseline, educational level of parents, family constitution, psychopathology of parents, and taking clustering in psychiatrist (team) into account.

Sensitivity analyses will be performed regarding risk of bias and missing data, in order to check the robustness of the results.

## Conclusion

The study contributes to implementation science in several ways. First, it examines transmural and interdisciplinary organizational changes (combined with professional education) as a method to enhance the implementation of a practice guideline. While the organizational changes are not entirely innovative (compare for instance programs for diagnosis of breast cancer), these are relatively new in child mental healthcare. Furthermore, studies on integrated care models tend to focus on health outcomes but provide little insight into mediating factors (e.g. changes in professional behavior). Secondly, it tests the impact of a potentially very efficient method of continued professional education—as only GPs with a referred ADHD patient receive online education. If effective and efficient, this could be a model for many other conditions—particularly those that are rare in a general practice population.

## Authors’ original submitted files for images

Below are the links to the authors’ original submitted files for images. Authors’ original file for figure 1
