# Supplementary material for: Improving patient care for attention deficit hyperactivity disorder in children by organizational redesign (Tornado program) and enhanced collaboration between psychiatry and general practice: a controlled before and after study
Source: Implement Sci. 2014 Oct 30;9:155. doi: 10.1186/s13012-014-0155-3 (PMC4219050; doi:10.1186/s13012-014-0155-3)
Supplement: Supplementary file 1 — Authors’ original file for figure 1 [file 13012_2014_155_MOESM1_ESM.docx]

Table 1: Participant flow, time points, questionnaires and respondents

|  | | | **Study group Tornado n=90** | | | **Control group Care As Usual n=90** | | |
| --- | --- | --- | --- | --- | --- | --- | --- | --- |
| **Timepoints** | **Child**  **(if >= 10yrs)** | **Parents/caregivers** | | **Professional (psychiatrist (T2) and general practitioner(T4, T7))** | **Child**  **(if >= 10yrs)** | | **Parents/caregivers** | **Professional (psychiatrist or pediatrician)** |
| **T1= referral** | HonosCA  Kidscreen  EQ-5D | ADHD-RS  HonosCA  Kidscreen  EQ-5D  Extra questions* | |  | HonosCA  Kidscreen  EQ-5D | | ADHD-RS  HonosCA  Kidscreen  EQ-5D  Extra questions* |  |
| **T2= Intake, start diagnostic process** | HonosCA  Kidscreen | ADHD-RS  HonosCA  Kidscreen  EQ-5D | | ADHD-RS  CGI  HonosCA | HonosCA  Kidscreen | | ADHD-RS  HonosCA  Kidscreen  EQ-5D | ADHD-RS  HonosCA  CGI |
| **T3= Consultation, end of diagnostic process** | GGZ-Thermometer | GGZ-Thermometer | |  | GGZ-Thermometer | | GGZ-Thermometer |  |
| **T4= Start pharmacotherapy** | HonosCA  Kidscreen | ADHD-RS  HonosCA  Kidscreen  Tic-P  EQ-5D | | ADHD-RS  CGI | HonosCA  Kidscreen | | ADHD-RS  HonosCA  Kidscreen  Tic-P  EQ-5D | ADHD-RS  HonosCA  CGI |
| **T5= 6 weeks after start pharmacotherapy** | HonosCA  Kidscreen  Morisky | ADHD-RS  HonosCA  Kidscreen  Morisky  EQ-5D | |  | HonosCA  Kidscreen  Morisky | | ADHD-RS  HonosCA  Kidscreen  Morisky  EQ-5D |  |
| **T6=9 months after T1** | Morisky  HonosCa  Kidscreen  EQ-5D | Morisky  HonosCa  Kidscreen  EQ-5D | |  | Morisky  HonosCa  Kidscreen  EQ-5D | | Morisky  HonosCa  Kidscreen  EQ-5D |  |
| **T7= 1 year after T0** | HonosCA  Kidscreen  EQ-5D  Morisky  GGZ-Thermometer | ADHD-RS  HonosCA  Kidscreen  Tic-P  EQ-5D  Morisky  GGZ-Thermometer | | ADHD-RS  HonosCA  CGI | HonosCA  Kidscreen  EQ-5D  Morisky  GGZ-Thermometer | | ADHD-RS  HonosCA  Kidscreen  Tic-P  EQ-5D  Morisky  GGZ-Thermometer | ADHD-RS  HonosCA  CGI |

*sex, family constitution, education level parents
